# The fragile X proteins’ enigma: to be or not to be nucleolar

**DOI:** 10.3389/fcell.2024.1448209

**Published:** 2024-08-02

**Authors:** Edouard W. Khandjian, Tom Moss, Timothy M. Rose, Claude Robert, Laetitia Davidovic

**Affiliations:** ^1^ Département de Psychiatrie et de Neurosciences, Faculté de Médecine, Université Laval, et Centre de Recherche Cervo, Québec, QC, Canada; ^2^ Département de Biologie Moléculaire, Biochimie Médicale et Pathologie, et Centre de Recherche sur le Cancer, Axe Oncologie, Centre de Recherche du CHUQ, Université Laval, Québec, QC, Canada; ^3^ Department of Pediatrics, University of Washington, Seattle, WA, United States; ^4^ Département des Sciences Animales, Université Laval, Québec, QC, Canada; ^5^ Centre National de la Recherche Scientifique UMR7275, Institut de Pharmacologie Moléculaire et Cellulaire, Inserm U1318, Université Côte d’Azur, Valbonne, France

**Keywords:** fragile x, nucleoli, Cajal body, translation, RNA-binding protein

## The context

The Fragile X Messenger Ribonucleoprotein (FMRP, previously referred to as Fragile Mental Retardation Protein, see comments in Khandjian et al. (2022) is an RNA-binding protein whose mutations or absence cause Fragile X Syndrome (FXS). FMRP is mainly found in the cytoplasm and has been implicated in translation regulation. It has also been suggested to be present in the nucleolus and have a function in ribosome biogenesis. Here we wish to critically survey the data supporting this potentially important secondary function.

FMRP is the archetype of a family of cytoplasmic RNA-binding proteins that includes the Fragile X related proteins FXR1P and FXR2P. The primary transcripts of the *FMR1* and *FXR1*, genes undergo alternative splicing processes (Ashley et al., 1993; Verkerk et al., 1993; Sittler et al., 1996; Kirkpatrick et al., 1999), resulting in multiple protein isoforms. Twelve FMRP isoforms have been detected, nine for FXR1P and one for FXR2P. Members of the Fragile X protein family are widely expressed in human tissues and in other mammals, albeit at varying levels, and expression of their isoforms is subtly choreographed (Davidovic et al., 2006a). FMRP is highly abundant in brain and testis but is absent in striated muscles. FXR1P is strongly expressed in striated muscle and testis and lower levels are detected in the brain. FXR2P expression remains almost constant in all organs and tissues. While the *FMR1* gene is present on chromosome X, *FXR1* and *FXR2* are autosomal genes present on chromosome 12 and 17, respectively.

In humans, mutations in the *FMR1* gene are the cause of FXS, a neurodevelopmental disorder that is characterized by development delay, intellectual disability, and in some cases autism spectrum disorders. FXS clinical presentation is highly heterogenous. FXS also affects peripheral tissues with patients exhibiting large everted ears, long face, increased cranial circumference, hypotonia, hyperlaxity of ligaments and macroorchidism (Hagerman et al., 2017). The most prevailing hypothesis regarding the physiopathology of FXS is that the absence of functional FMRP causes dysregulation of translation (Bassell and Warren, 2008; Darnell, 2011; Richter and Zhao, 2021). FXR1P is essential for muscle development (Mientjes et al., 2004; Huot et al., 2005) and recessive mutations in the muscle specific long isoform of FXR1P cause congenital multi-minicore myopathy in human and mice (Estañ et al., 2019), possibly by altering translation since FXR1P has been involved in translation regulation (Garnon et al., 2005; Huot et al., 2005; Vasudevan and Steitz, 2007). FXR2P is the least studied among the FXR proteins and, although it is also associated with the translational apparatus (Corbin et al., 1997), its role in translation regulation has not yet been formally determined.

**FIGURE 1:**
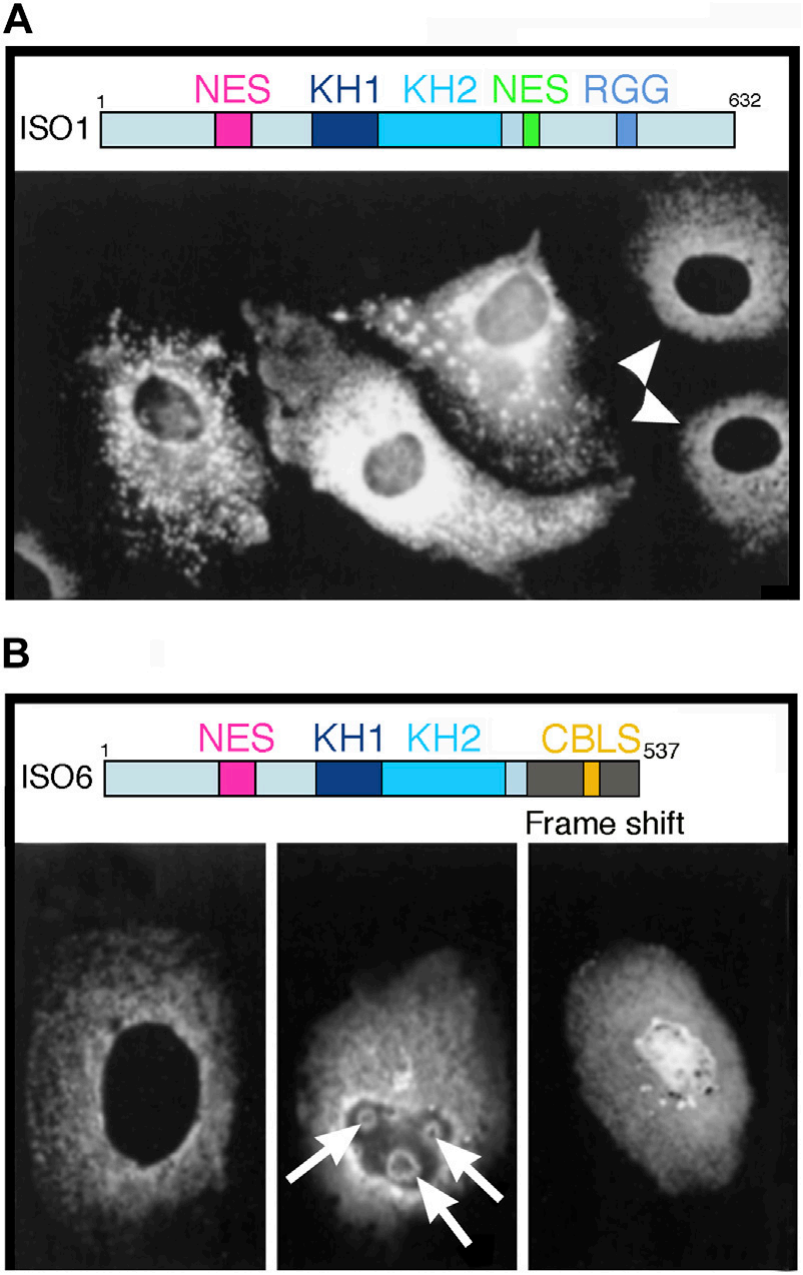
Overexpression of two FMRP isoforms induce unusual structures in Cos-1 transfected cells. **(A)** Overexpression of full length FMRP Iso1. Shown here are three different levels of expression in transfected cells as compared to endogenous expression in two untransfected cells indicated by white arrow heads. Note the aggregates and granules detected in the cytoplasm of transfected cells. **(B)** Overexpression of minor FMRP ISO6. Staining of endogenous FMRP in a non-transfected cells is shown in the left panel. Nuclear localization of exogenous FMRP ISO6 as diffuse nuclear repartition or as round shaped structures in the nucleolus (white arrows). Reproduced from (24) with permission from Biochemistry and Cellular Biology. Shown above each panel are the maps of the ISO1 full-length and the ISO6 isoform. Note the NLS, as well as the KH1 and KH2 domains in both isoforms.

The overall structure of the three proteins is very similar and FXR1P and FXR2P share 86% amino acid identity with FMRP in the central region, and 70% over the N-terminal region. All three proteins contain similar functional RNA-binding domains: two central KH domains (KH1 and KH2) and a C-terminal RGG domain. In addition, while the three proteins exhibit N-terminal nuclear localization signals (NLS) and a nuclear export signals (NES) between the KH and RGG domain (see maps in Figure 1) the main isoforms are exclusively detected in the cytoplasm. The C-termini of the three proteins are highly divergent and Tamanini et al. (2000) reported a nucleolar localization signal (NoLS) in FXR1P and FXR2P, which is absent in FMRP (Tamanini et al., 2000). However, some studies have challenged this view, and the issue of nucleolar localization of FMRP is the subject of the present opinion.

## FMRP, to be or not to be nucleolar?

Shortly after the detection of endogenous FMRP associated with the translation apparatus (Khandjian et al., 1996), a follow-up study reported the sub-cellular localization of FMRP based on its overexpression after transfection with a cDNA expression vector (Willemsen et al., 1996). Using this approach, the authors detected the presence of FMRP in large cytoplasmic aggregates and even larger aggregates in the nucleolus. It is important to note that the transfection assays were performed with a vector under the control of the strong SV40 promoter, and FMRP localization was analyzed at 72 h, an astoundingly long-time post-transfection. The likelihood that the conclusions on FMRP subcellular distribution drawn in this study by Willemsen et al. (1996) are generalized to endogenous FMRP is at best questionable since overexpression most probably overloaded the cellular machinery, resulting in the artifactual localization of FMRP in both the nucleus and the nucleolus (see below).

More recently, Taha et al. (Taha et al., 2014; Taha and Ahmadian, 2024) reported that endogenous FMRP can in fact be detected in the nucleolus and suggested that cytoplasmic-nucleolar shuttling is an important aspect of FMRP function. However, it is questionable whether the immunofluorescence images presented in Figure 1B of their paper support nucleolar localization of endogenous FMRP, as the fluorescence signal is not clearly distinguishable from background, nor does it clearly colocalize with nucleolin, the nucleolar protein used as marker (even when magnified at ×500). Their choice to use the ribosomal protein RLR0 as a nucleolar marker was not judicious in the context of the nucleolus, since this acidic protein is present only in cytoplasmic ribosomes. They also found that an exogenously expressed truncated fragment of FMRP, containing the N-terminal domains, but lacking the NES, was cytoplasmic [as shown in Figure 4E of their article (Taha et al., 2014)], in clear contrast with previous reports showing nuclear localization for this type of constructs (Devys et al., 1993; Bardoni et al., 1997). Based on sequence analysis, Taha et al. claimed that FMRP possesses three previously unidentified NoLS motifs in its C-terminal domain. NoLS3: 480HGRRGPGY489, NoLS2: 526LRRGDGRRRGG536, and NoLS1: 612NQKKEKPD619, which all map to different C-terminal exons (Taha et al., 2014). While the authors conclude that these motifs in FMRP are partially conserved with the previously detected NoLS motifs in FXR1P and FXR2P (Tamanini et al., 2000) and show motifs aligned, (see Figure 4D of their article reference Taha et al. (2014), the FMRP and FXR1P/FXR2P motifs appear unrelated since they show minimal sequence homology and are derived from distinct exonic sequences.

Importantly in the present context, a study on a FXS patient revealed a new mutation in the *FMR1* gene (Okray et al., 2015). In this patient, a Guanine (G) insertion in exon 15, at position 33020 in the gene was identified. This mutation alters the open reading frame creating a short C-terminal sequence, followed by a stop codon. This results in a mutated FMRP lacking the NES and the RGG domain similarly to the nuclear FMRP ISO6 (see below). In transfection assays using an expression vector containing the cDNA of the truncated mutant, this FMRP localizes exclusively to the nucleolus although this truncated protein does not contain the sequences of the hypothetical nucleolar localization signal (NoLS) published by Taha et al. (2014).

In support of their claims of a nucleolar localization of FMRP, Taha et al. (2014) also analyzed the presence of FMRP in subcellular organelles including nucleoli and in immunoprecipitates and reported that FMRP was as abundant in the nucleolus as in the cytosolic fractions. However, the experimental procedure used to obtain these organelles is highly questionable as it involved nuclear disruption by gentle homogenization with a Balch homogenizer. Well-established cellular fractionation procedures all agree that nucleoli extraction requires nuclei disruption by sonification to share the viscous chromatin and free the organelles (see seminal work, for instance from Harris Busch, Jean-Pierre Zalta, in the 60’s). Also, there is no universal method to purify all cell components based on a single cell lysis technique such as the one used by Taha et al. (2014). They also claimed that FMRP is associated with mitochondria without controlling for the possible contamination with the rough endoplasmic reticulum that contains polyribosomes and by consequence FMRP. Based on these technical considerations, we believe there are serious concerns regarding the conclusions drawn by Taha et al. (Taha et al., 2014; Taha and Ahmadian, 2024), which more than likely stem from over enthusiastic interpretation of the images and the use of inadequate experimental procedures. Finally, they concluded their study by transfection assays using a pcDNA3 eukaryotic expression vector in which *FMR1* full-length cDNA as well several truncated forms were expressed under the strong cytomegalovirus (CMV) promoter. The atypically shape of the nucleoli stained with anti-nucleolin antibodies (their Figure 4 in Taha et al. (2014), suggests that the transfected cells were likely undergoing distress, once again questioning the conclusions drawn.

## Avoiding traps to solve the problem in future studies

Although the expression of exogenous proteins via transfection assays is a convenient approach to study subcellular localization, we posit that results obtained may not necessarily be generalizable to the native endogenous protein that is subjected to natural regulation of its expression level that is tightly linked to the cellular state (e.g., mitotic, quiescent, metabolic, stressed …). Based on our long-standing experience, overexpressing of FMRP at high levels and for extended periods of time results in unusual if not atypical images. Our long-standing experience related to the use of exogenous FMRP support this. In the late 1990’s, we transfected Cos-3 cells (Khandjian, 1999) independently with two *FMR1* cDNA variants under the control of the simian virus (SV40) promotor in the pTL1 vector to allow expression of the longest FMRP isoform (ISO1, 632 aa) and a minor isoform (ISO6, 537 aa) in which a frame shift at aa 425 in the C-terminus causes a premature truncation of the protein and the absence of the NES and RGG domains (Dury et al., 2013). After 24 h of transfection, cells were fixed and stained with the mAb1C3 monoclonal antibody that recognizes the constitutive N-terminal part (aa 66–112) of FMRP present in all isoforms. In untransfected cells (white arrow heads in Figure 1A), FMRP exhibited its classical, cytoplasmic staining while nuclear staining was absent. In contrast, cells overexpressing ISO1 exhibit strong reactive aggregates and granules in the cytoplasm. Subsequently, we have shown that these granules correspond to stress granules that sequester mRNA and many other RNA-binding proteins (Mazroui et al., 2002). These stress granules form in response to cellular stress (heat shock, hypoxia, arsenite, UVC, etc.) and maintain translational repression until the stress is relieved.

Regarding FMRP ISO6, half of the transfected cells exhibited a strong ring-shape staining surrounding the nucleolus, while the other half exhibited a punctuate staining in the nucleus (Khandjian, 1999). However, in the latter case, the nucleoli appeared irregular in shape (Figure 1B). Being truncated, FMRP ISO6 isoform only contains the N-terminal and C-central regions and lacks the three putative C-terminal NoLS motifs described by Taha et al. (2014) that, in contrast to the early data, were found by these authors to be distributed uniformly throughout nucleus and cytoplasm (their Figure 1b in Taha et al. (2014)). Furthermore, the ring-shaped nucleolar structures which we observed and are absent under normal growth conditions, are reminiscent of the nucleolar detention centers (DC) in which proteins are temporarily detained in response to cellular stresses (Audas et al., 2012a; Audas et al., 2012b; Jacob et al., 2013). Such structural remodeling leaves nucleoli temporarily unable to sustain their primary function of ribosomal biogenesis until stress is relieved. In a certain way, formation of DC could be the nucleolar counterpart of cytoplasmic stress granules formation (see above).

Bearing all this in mind, we have paid attention in our work to limiting FMRP transient expression of FMRP to less than 18 h, in contrast to many studies in the field that used up to 72 h. In this context, the minor FMRP ISO6 clearly localizes to nucleoli (Davidovic et al., 2006b) and Cajal Bodies (Dury et al., 2013). These two organelles have been previously described notably a century ago (Garcia-Lopez et al., 2010) to be in direct physical contact. Much more recently, it was observed in HeLa cells that Cajal Bodies shuttle in and out of nucleoli (Gall, 2000; Platani et al., 2000; Cioce and Lamond, 2005). Our results are in line with this model.

Taking these considerations together, we suggest that there is a lack of convincing literature-based evidence to support a nucleolar role for full-length FMRP ISO1 in ribosome biogenesis. Rather we suggest that the artificial transient overexpression conditions used in most studies, disturbs cellular homeostasis and leads to FMRP mis-localization. We hope that this opinion will serve as a reference for future studies exploring the nuclear and more specifically nucleolar roles of FMRP.
